# Supplementary figures and images for: Assembly of the mitochondrial outer membrane module of the trypanosomal tripartite attachment complex
Source: PLoS Pathog. 2025 Sep 9;21(9):e1013506. doi: 10.1371/journal.ppat.1013506 (PMC12440218; doi:10.1371/journal.ppat.1013506)

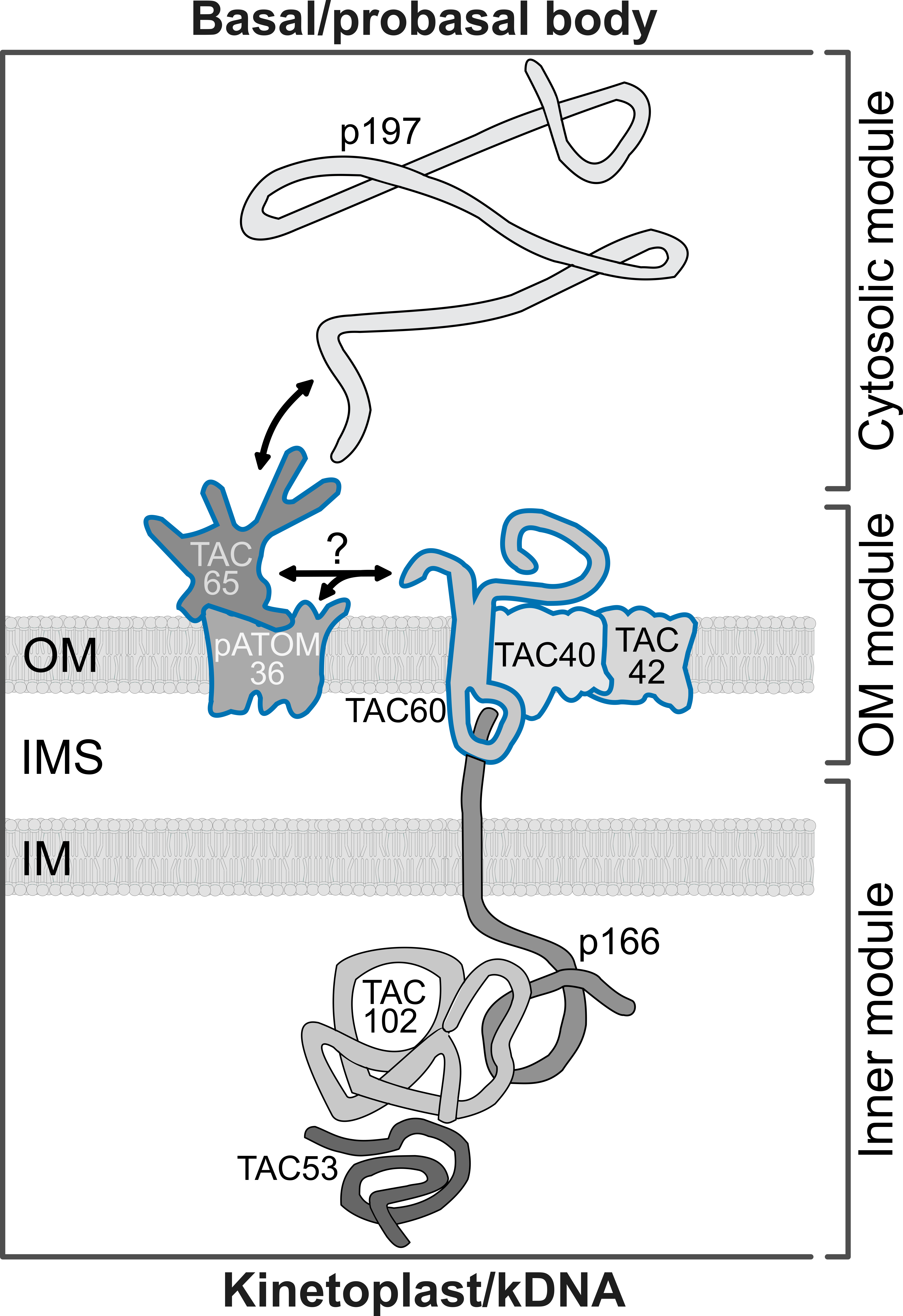

Supplement: S1 Fig — On the molecular level the TAC can be divided into three modules: (i) the cytosolic TAC module, (ii) the OM TAC module, and (iii) the inner TAC module. The cytosolic TAC module consists exclusively of p197, a protein anchored at the basal body which connects to an unknown domain of TAC65 at the OM. The OM TAC module contains the peripheral membrane protein (TAC65), two integral membrane proteins (pATOM36, TAC60) with α-helical transmembrane domains, and two β-barrel membrane proteins (TAC40, TAC42). TAC65 and pATOM36 interact, the same is the case for TAC40, TAC42, and TAC60. How the two groups of proteins interact with each other is unclear (?). TAC60 interacts with p166, an integral IM protein with a single α-helical transmembrane domain. As a part of the inner TAC module, p166 interacts with TAC102 in the mitochondrial matrix. TAC53 is the inner TAC module subunit that is most proximal to the kDNA. (TIF) [file ppat.1013506.s001.tif]

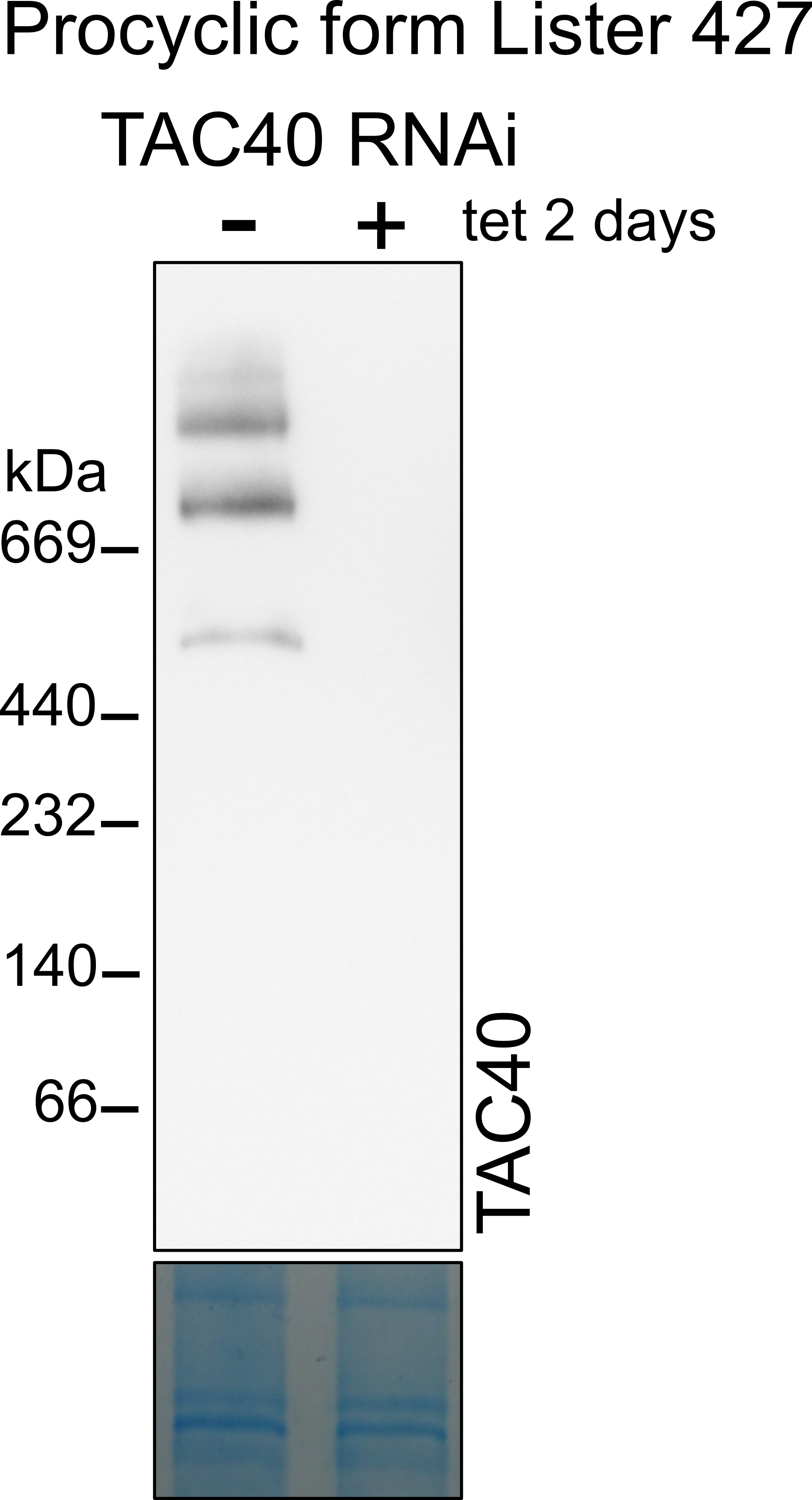

Supplement: S2 Fig — Immunoblot of a BN-PAGE experiment with an uninduced (-tet) and TAC40 RNAi induced (+tet 2 days) procyclic T. brucei cell line probed with the TAC40 antibody. The positions of marker proteins with their size in kDa are indicated on the left. Coomassie blue-stained gel sections (bottom) serve as loading controls. (TIF) [file ppat.1013506.s002.tif]

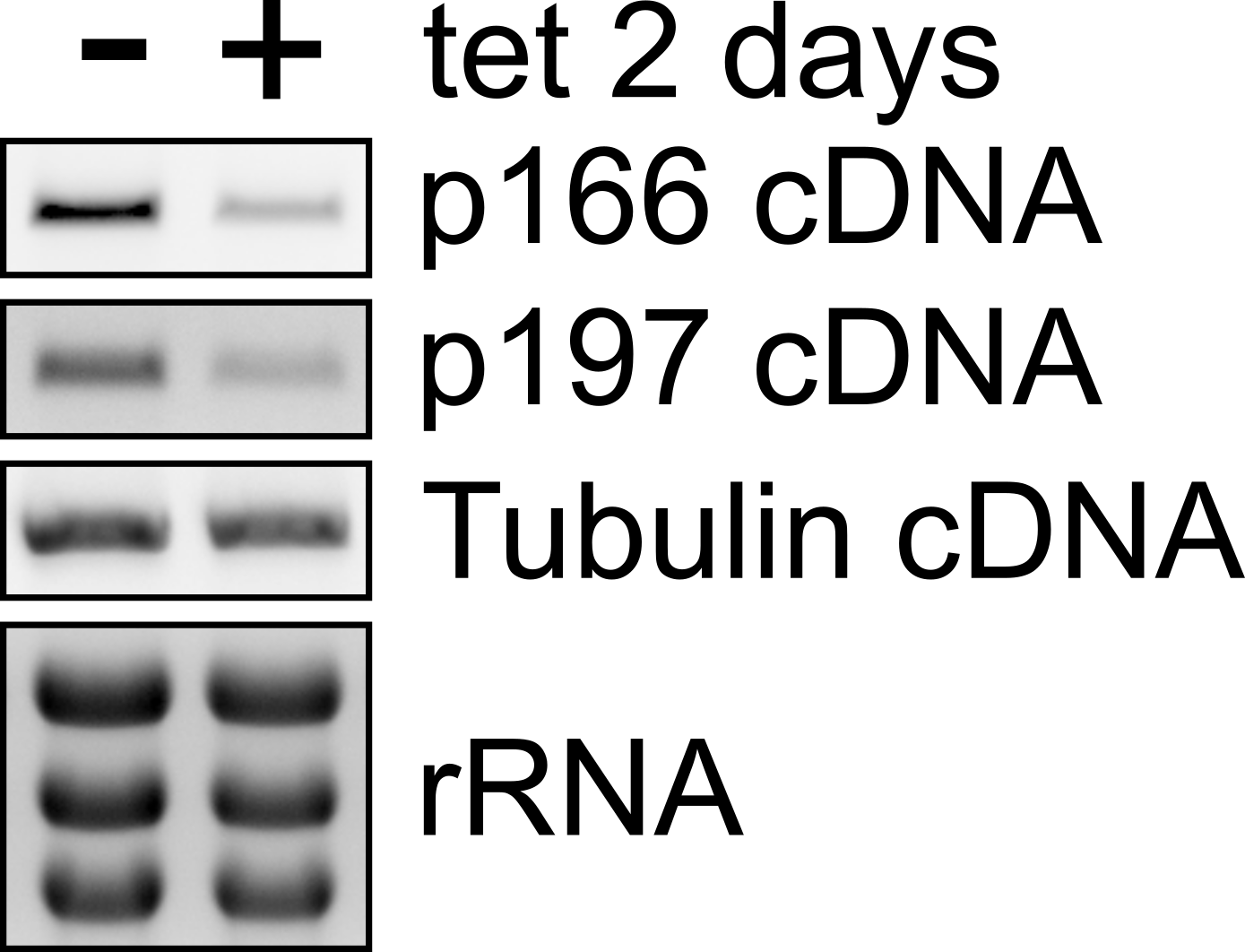

Supplement: S3 Fig — Ethidium bromide-stained agarose gels of PCR amplified cDNA segments corresponding to p166, p197, and tubulin mRNAs, as well as non-amplified cytosolic rRNA of the uninduced and induced p166/p197 double RNAi cell line. Tubulin cDNA and rRNA signals serve as loading controls. (TIF) [file ppat.1013506.s003.tif]

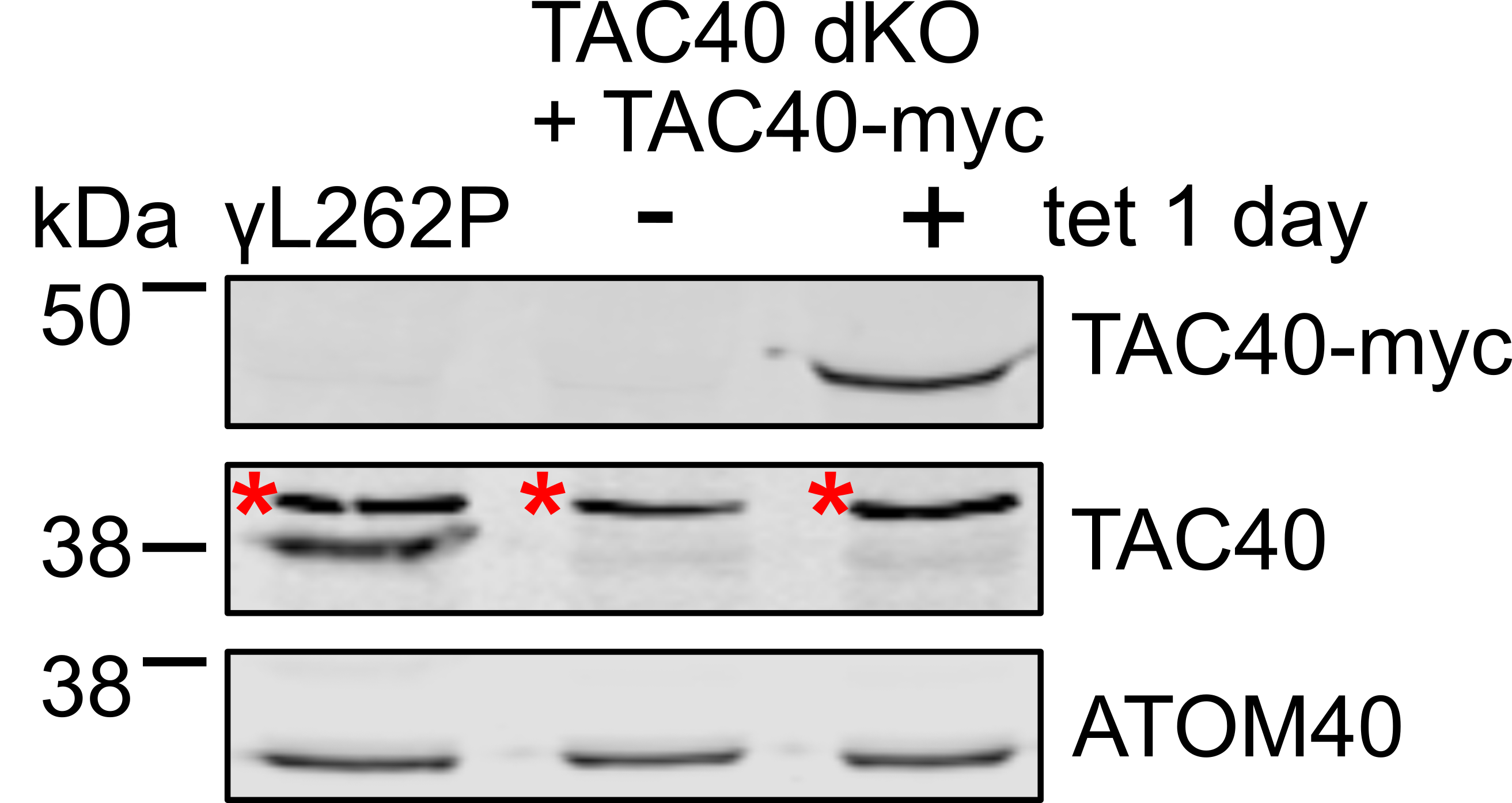

Supplement: S4 Fig — Immunoblots comparing whole cell protein levels of TAC40-myc (top panel), TAC40 (middle panel), and ATOM40 (bottom panel). The parent γL262P BSF cell line (left lane) and TAC40 dKO γL262P BSF uninduced (middle lane) and induced (right lane) for ectopic expression of TAC40-myc were analyzed. The ATOM40 signals serve as a loading control. Numbers on the left indicate protein size markers in kDa. Asterisk, unspecific band recognized by the polyclonal TAC40 antiserum. (TIF) [file ppat.1013506.s004.tif]

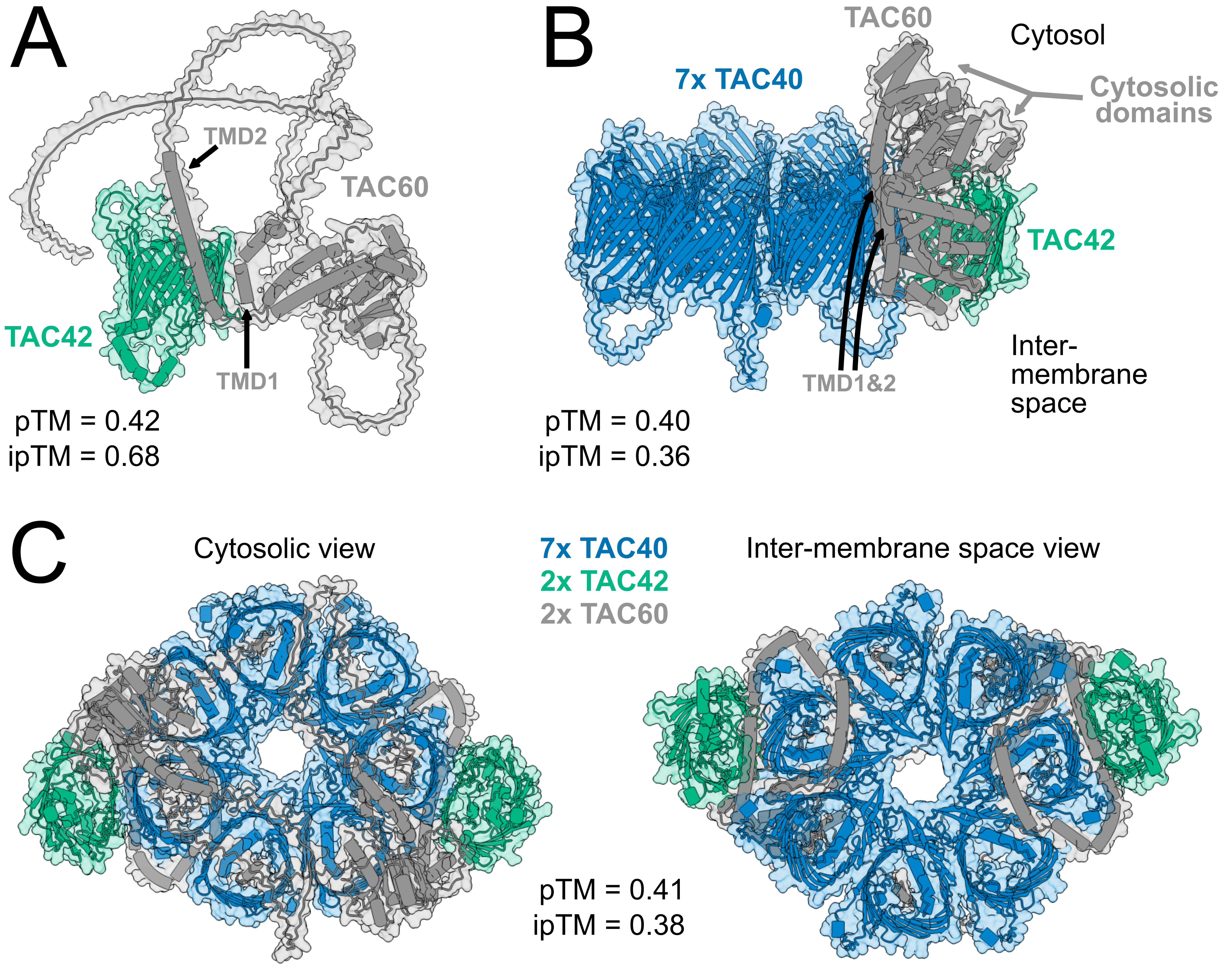

Supplement: S5 Fig — (A) Model of an AlphaFold3 structure prediction for a TAC42/TAC60 dimer shown from the side of the membrane. TMD: α-helical transmembrane domain; pTM: predicted template modelling score; ipTM: interference pTM. (B) Model of an AlphaFold3 structure prediction for a complex possibly matching the ~ 770 kDa TAC40/TAC42/TAC60 subcomplex shown from the side of the membrane. (C) Model of an AlphaFold3 structure predictions for a complex possibly matching the ~ 920 kDa TAC40/TAC42/TAC60 subcomplex shown from the cytosolic (left) and the inter-membrane space (right) side. (TIF) [file ppat.1013506.s005.tif]

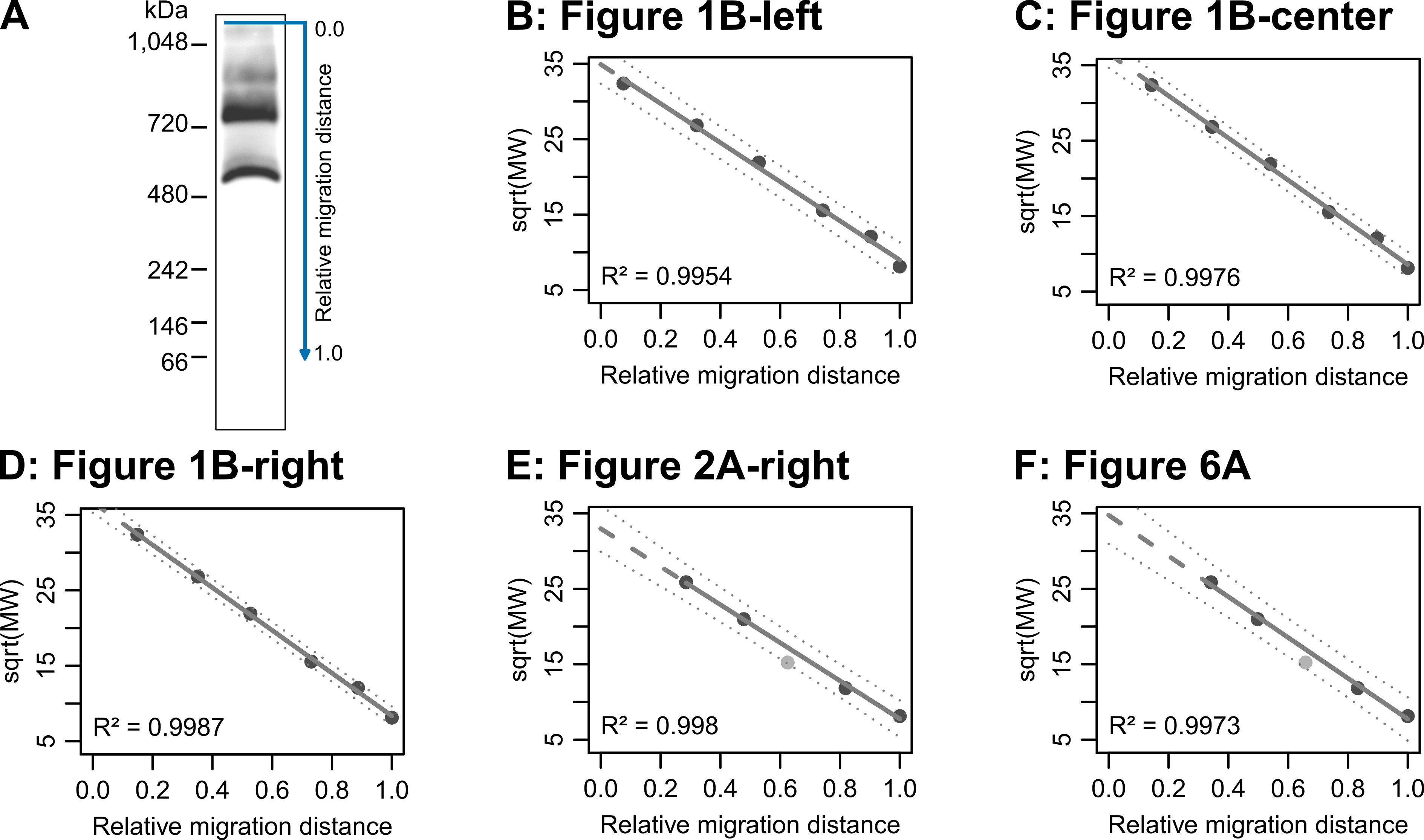

Supplement: S6 Fig — (A) Definition of “relative migration distance”: distance between the upper edge of the gel and the band of interest divided by the distance between the upper edge of the gel and the lowest molecular weight marker. The shown lane is identical to the panel in Fig 1B (left). (B-F) Graphs depicting the linear models of the relative migration distance of marker proteins versus the square root (sqrt) of the molecular weight in kDa. Dark gray datapoints represent marker protein data used for the linear models. Light gray datapoints in (E) and (F) show marker measurements which were omitted for calculation of linear models. The solid and dashed gray lines show the inter- and extrapolated linear models, respectively. Dotted lines show the 90% prediction confidence intervals. The coefficient of determination (R2) of the linear model is shown at the bottom left of each graph. (TIF) [file ppat.1013506.s006.tif]

**Fig 2A**

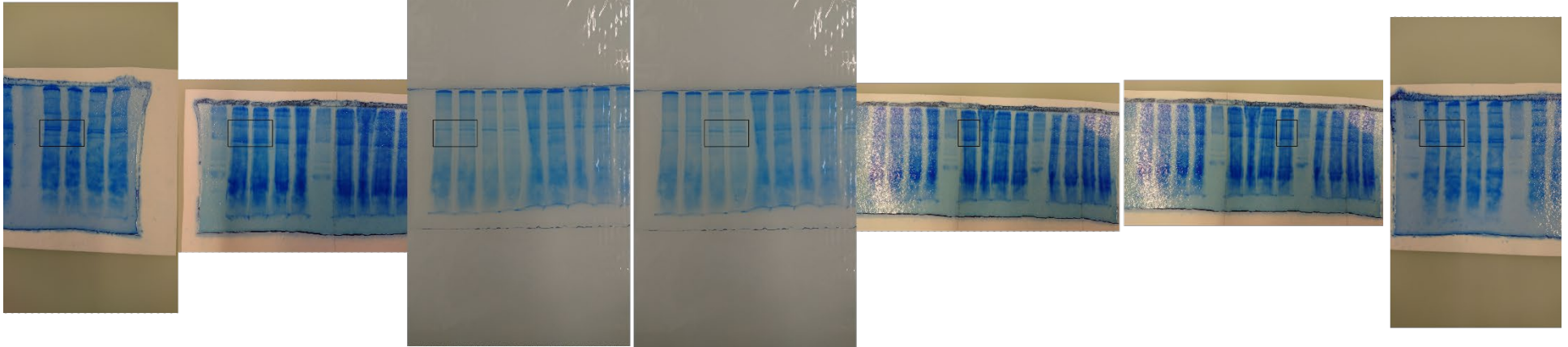

**Fig 2B**

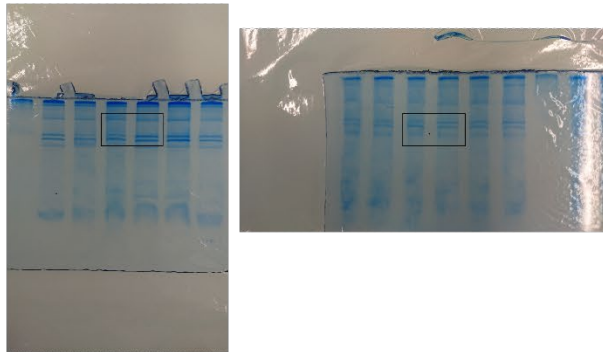

**Fig 2C**

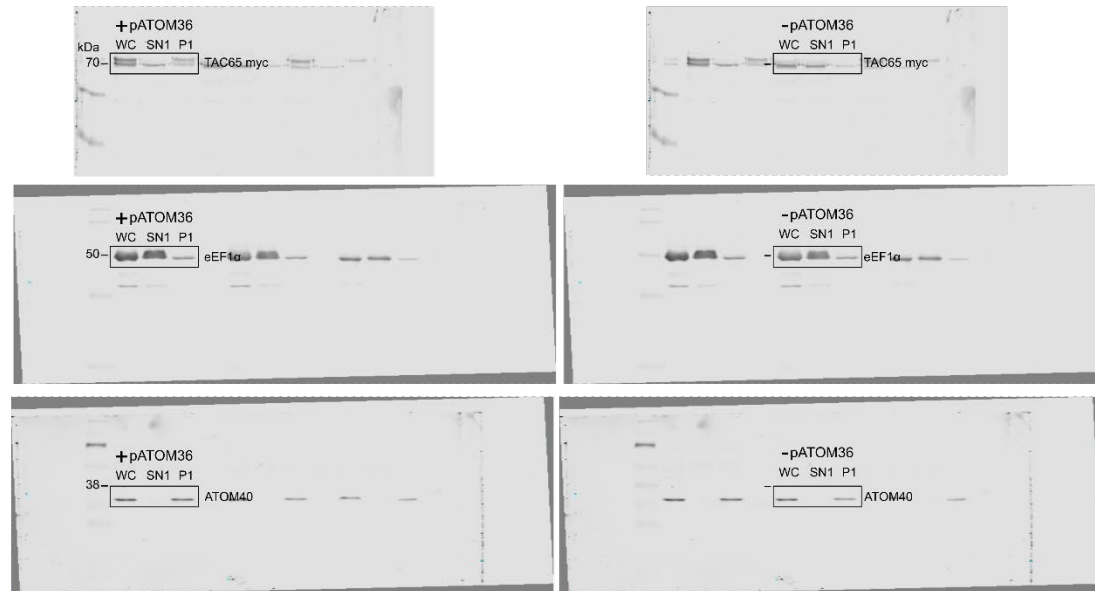

**Fig 3A**

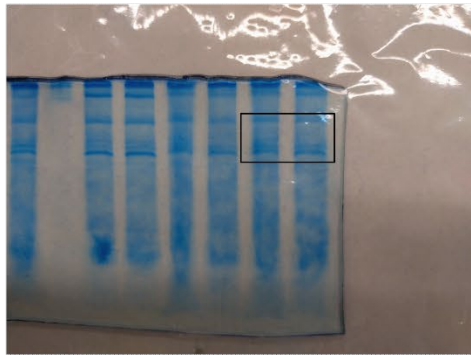

**Fig 3B**

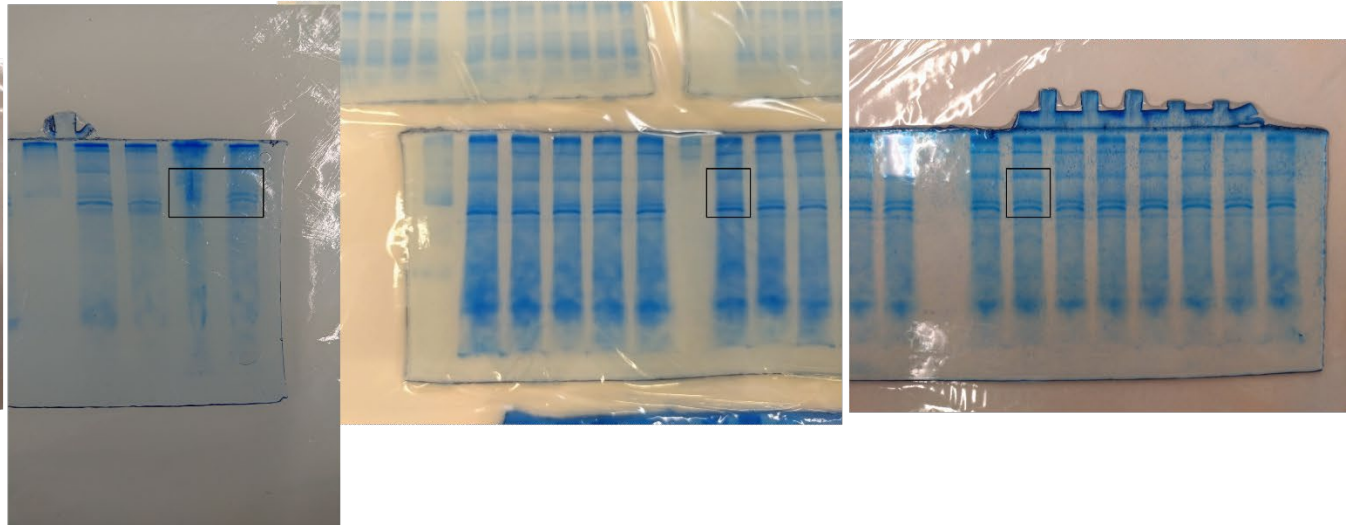

**Fig 4A**

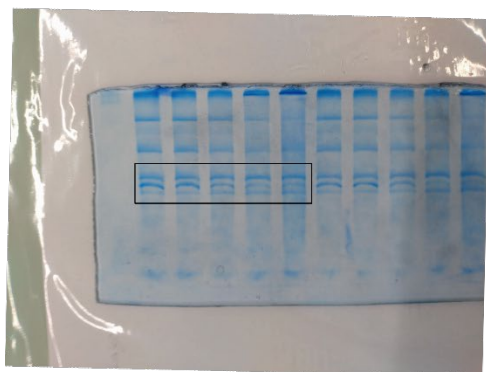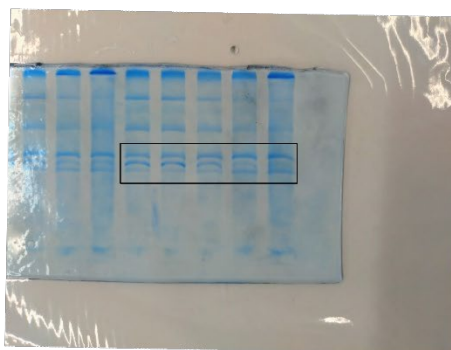

**Fig 4B**

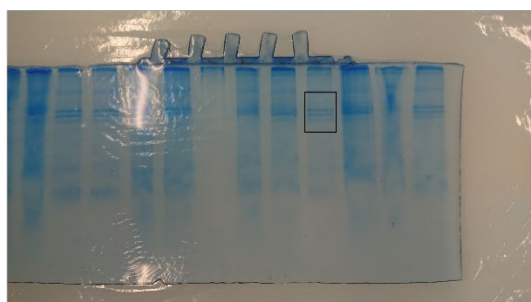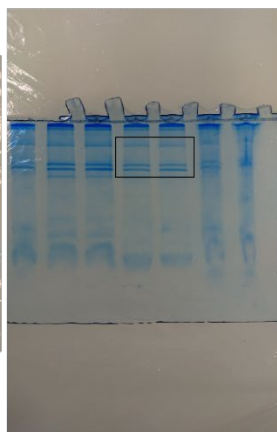

**Fig 4C**

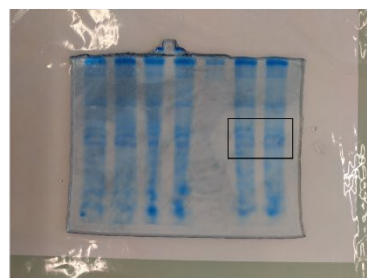

# Fig 5

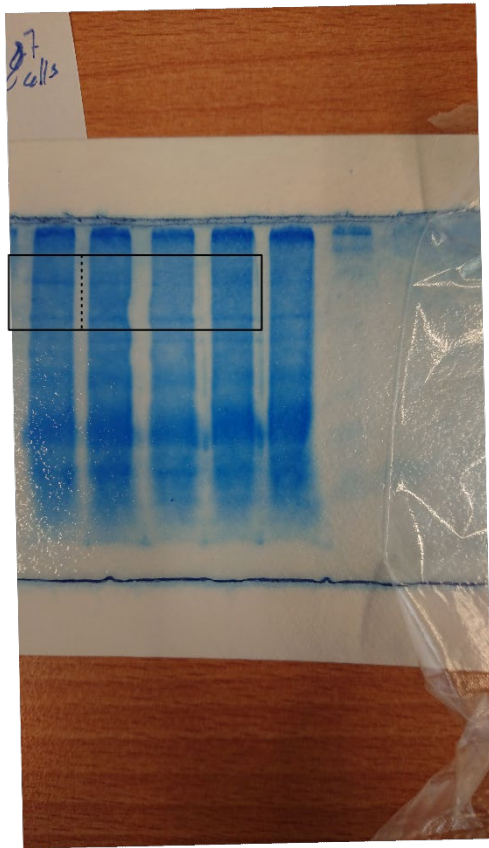

# Fig 6A

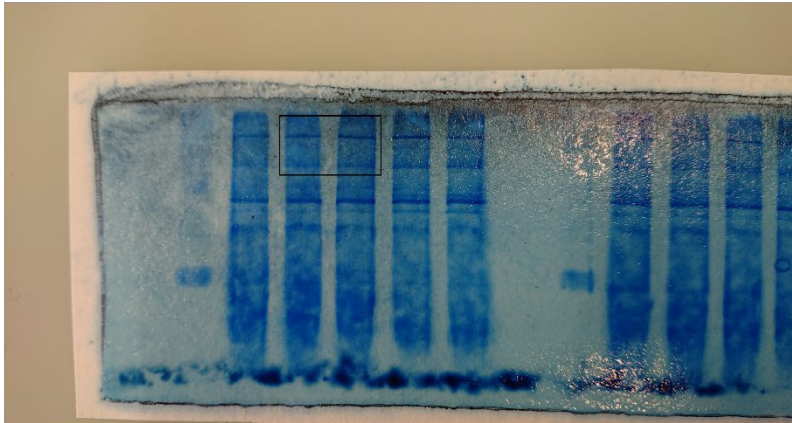

# Fig 6C

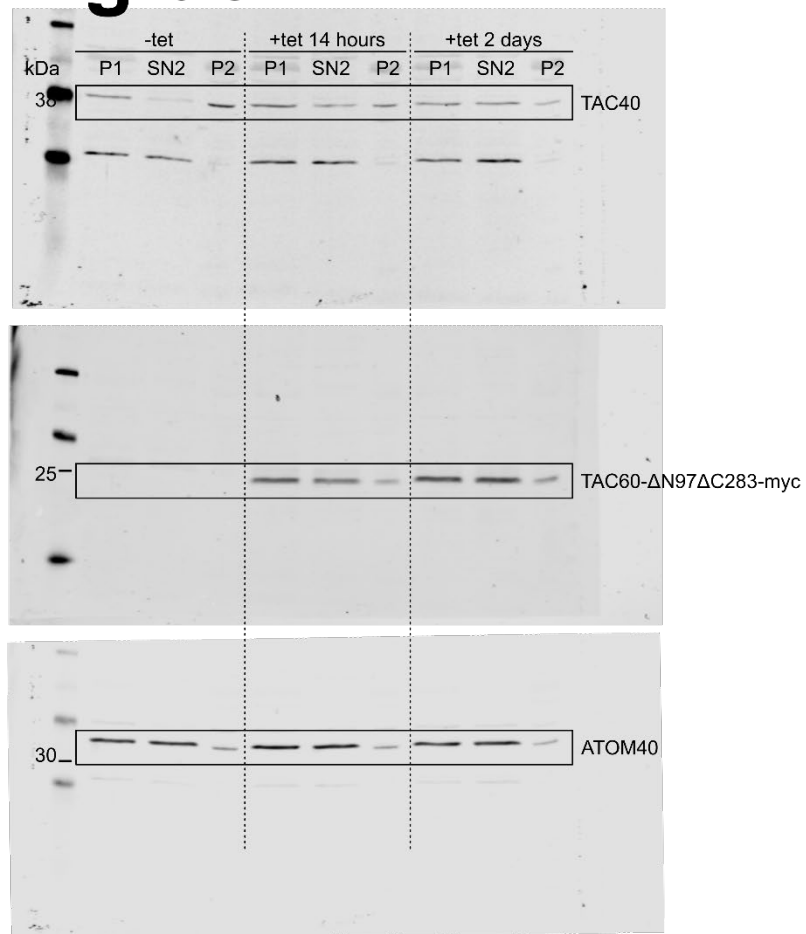

# Fig 7 B

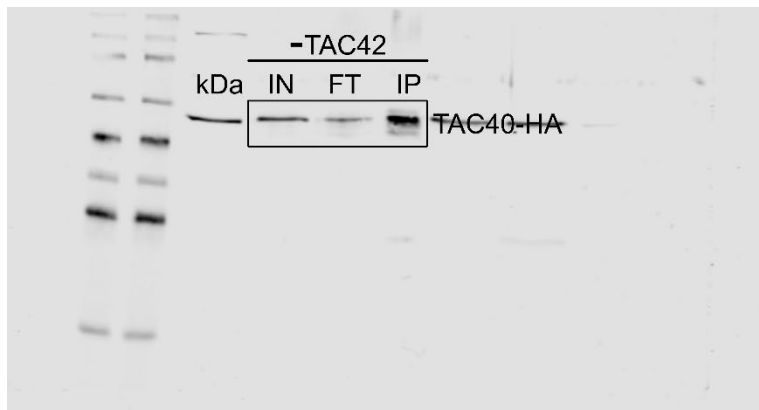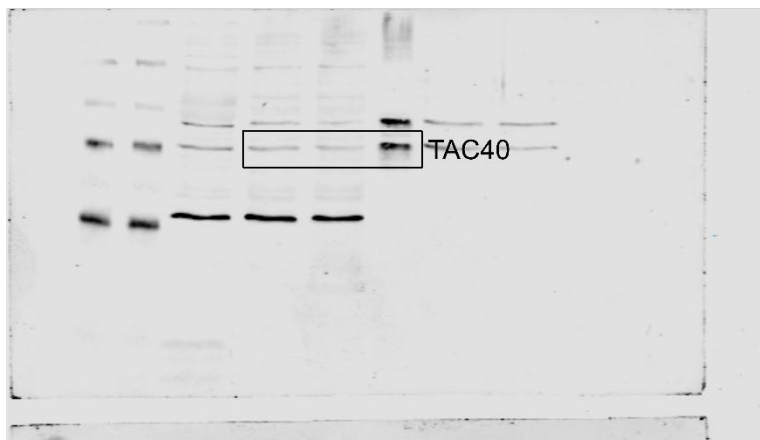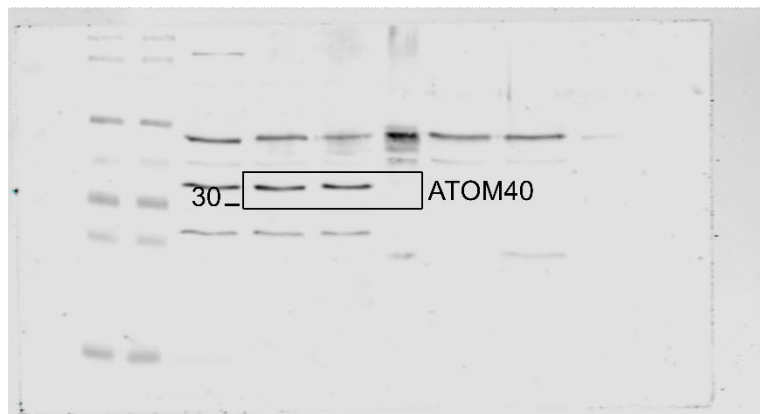

## S2 Fig

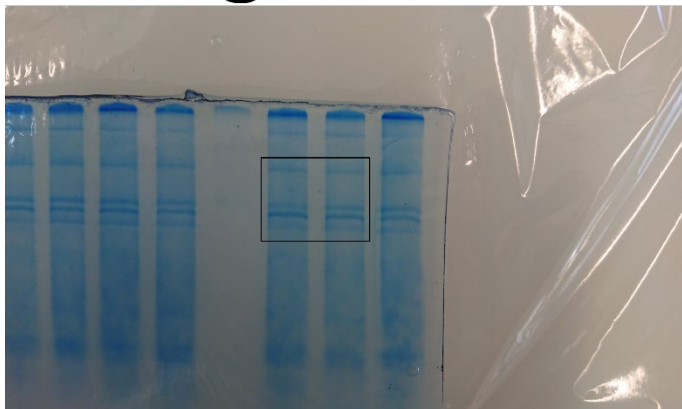

S3 Fig

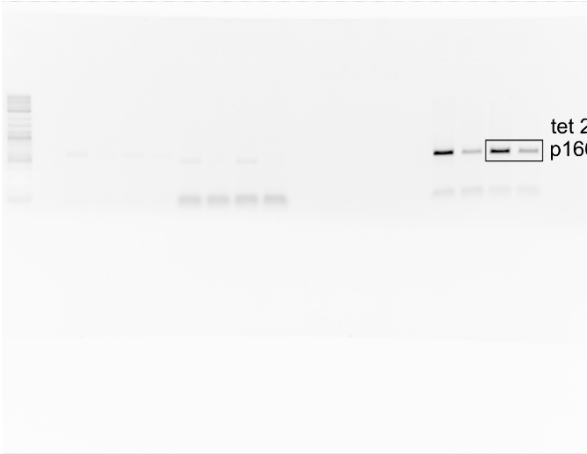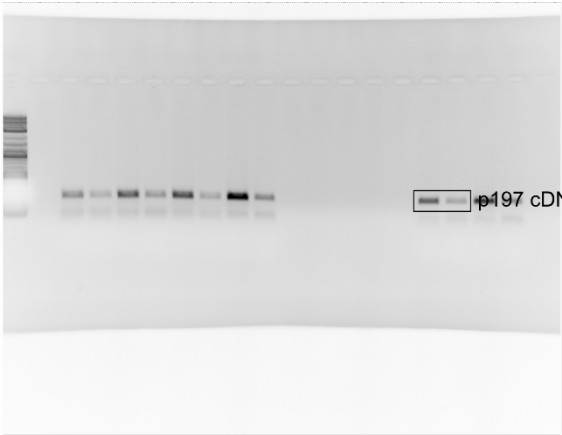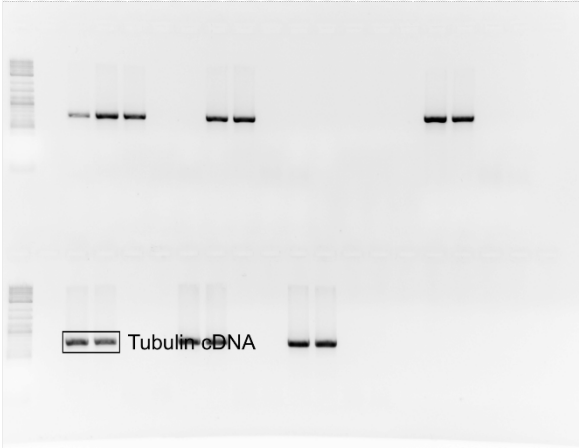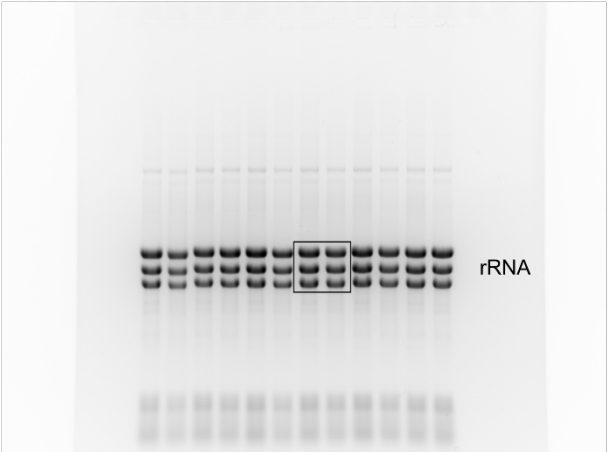

# S4 Fig

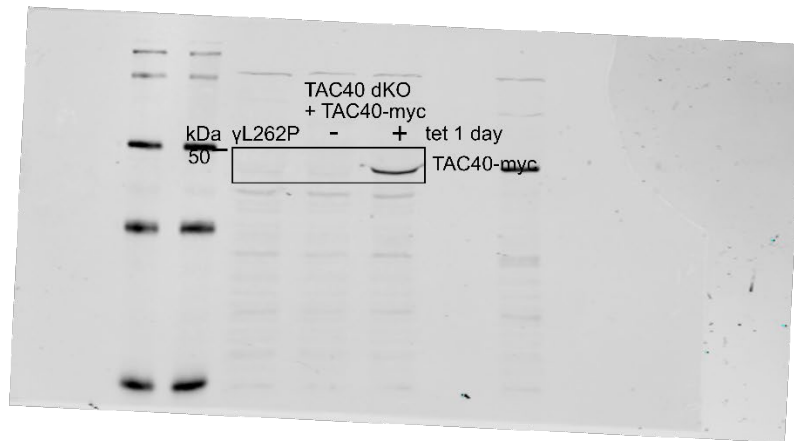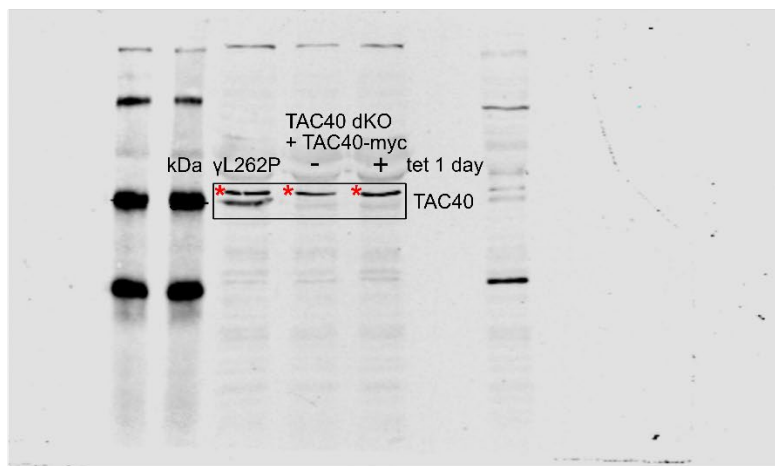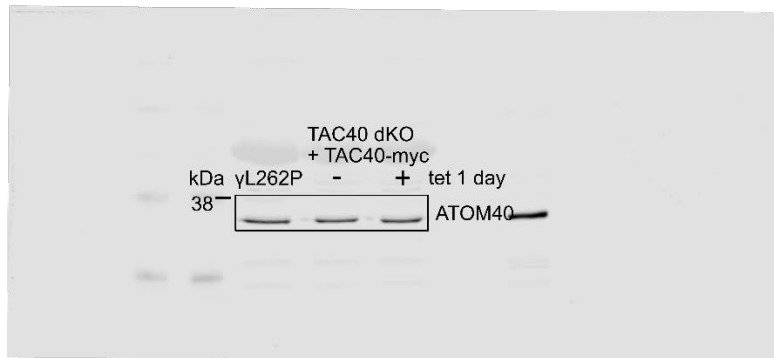

Supplement: S1 Raw images — (PDF) [file ppat.1013506.s008.pdf]
