# Supplementary material for: Assembly of the mitochondrial outer membrane module of the trypanosomal tripartite attachment complex
Source: PLoS Pathog. 2025 Sep 9;21(9):e1013506. doi: 10.1371/journal.ppat.1013506 (PMC12440218; doi:10.1371/journal.ppat.1013506)
Supplement: Attachment — Submitted filename: Philip-PPath-Rebuttal.docx [file ppat.1013506.s010.docx]

***Reviewers' Comments:***

***Reviewer's Responses to Questions***

***Part I - Summary***

***Reviewer #1:*** *In this study, Stettler et al. examined the assembly of TAC modules that reside within the mitochondrial outer membrane, using a combination of blue native PAGE and RNAi. Based on the results, the authors proposed a step wise assembly pathway from TAC40 oligomers, to larger TAC42-TAC40- TAC60 subcomplexes, and subsequent assembly with pATOM36-TAC65 subcomplex to form the OM TAC module. A detailed, accurate description of the OM-TAC module assembly is an important step to understand TAC biogenesis. Considering the limitation of BN-PAGE/immunoblots in subcomplex fractionation and detection (detailed below), additional approaches and controls are recommended to validate some of the key observations made in this study.*

***Reviewer #2:*** *The manuscript by Stettler et al. investigates the composition of distinct subcomplexes formed by the tripartite attachment complex (TAC) components in Trypanosoma brucei. The authors identify detergent-soluble assemblies located in the outer membrane (OM) segment of the TAC. Their data suggest that OM TAC modules form three distinct subcomplexes: one containing TAC40, another with TAC42 and TAC60, and a separate subcomplex consisting of pATOM36 and TAC65. Through a combination of Blue Native PAGE (BNPAGE), RNA interference (RNAi)-mediated protein depletion, and restoration experiments in parasites lacking kinetoplast DNA (kDNA), the authors propose an assembly pathway that includes TAC40 oligomerization and subsequent binding events.
While the presented model aligns with the data, there are two main concerns regarding the robustness and functional significance of the findings.*

**Part II – Major Issues: Key Experiments Required for Acceptance**

**Reviewer #1*:***

*1. Fractionation of OM complexes by BN-PAGE. In this study, BN-PAGE was combined with immunoblottings to detect different OM-TAC subcomplexes. Although a powerful method to study native protein complexes, BN-PAGE does have limitations.*

*In Fig. 1A, in addition to the labelled 920, 770 and 535 subcomplexes, there are clearly other higher molecular weight bands (> 1,048kDa) in each of the blots for TAC40, TAC42-HA and TAC60-myc, suggesting the presence of higher order complexes containing all 3 subunits?*

All BN-PAGE gel lanes shown in Fig. 1A (Fig. 1B in the revised manuscript) show the entire gel/blot. It includes a small part of the gel region that contains the loading pocket which was poured on the top of the separating gradient gel. In these regions additional large complexes which contain our proteins of interest are observed that barely migrate into the gels. The problem is that these bands are not consistently detected, and that it is challenging to discriminate them. This is mentioned in the text (lines 206-208). Another reason to not consider these bands in our analysis was that the focus of our study is on early assembly complexes which have a lower molecular weight. These limitations are also mentioned in the discussion section, where it is stated that larger assembly intermediates likely exist but could not be characterized (lines 575-577).

*Besides, it was concluded that the 920 KDa subcomplex contained much less TAC40 protein than the other, smaller subcomplexes (lines 175-176). This is an ambiguous statement. While it is possible that the 920 KDa complex is present at lower abundance, it is also possible that the transfer of the larger complexes is less efficient during the blotting process, leading to less intense signals compared to smaller complexes. As such, even larger complexes may be present but not detectable on BN-PAGE.*

This is indeed possible. We clarified the statement, it now reads (lines 184-187):

“The bands corresponding to the ~535 and ~770 kDa subcomplexes contained similar amounts of TAC40, whereas the band corresponding to the ~920 kDa subcomplex contained much less of the protein, suggesting the subcomplex is less abundant. Alternatively, it is also possible that the blotting of the larger complexes is less efficient.”

We also removed the following sentence from the manuscript, as it may give misleading information regarding the amounts of the different subcomplexes.

“The two subcomplexes displayed similar signal intensities when probed for TAC60 whereas the ~920 kDa subcomplex gave a slightly less intense signal relative to the ~770 kDa subcomplex when probed for TAC42.”

*Considering the consistency issue often associated with BN-PAGE, and size-correlated protein transfer efficiency during blotting, it would be important to corroborate the BN-PAGE results with other complex fraction/detection methods that may be less affected by size. For example, size exclusion chromatography followed by mass spec or SDS-PAGE and immunoblots of the isolated subcomplexes, may help to verify the presence and relative abundance of different subcomplexes*

Size exclusion chromatography of the detergent soluble complexes would be very challenging because the assembly complexes are present at very low abundance only.

However, the same limitations do not necessarily apply to pulldown experiments which result in a concentration of the complexes. Indeed, previous publications from our lab included individual proteomic analyses of TAC40, TAC42, TAC60, TAC65 and pATOM36 SILAC pulldown experiments [36, 48]. Prompted by the reviewer comments we have now integrated all five data sets and quantitatively analyzed them together. This allowed the construction of an edge-weighted protein-protein interaction network visualizing the digitonin-soluble interactomes of TAC40, TAC42, TAC60, TAC65 and pATOM36 (new Fig. 1A). – For a detailed discussion of the results and the modification that were made to the manuscript, see response to the first comment of reviewer 2 in this section (Part II, major issues).

*2. It appears that the tagged TAC42 and TAC60 subunits are introduced into different cell lines and thus the BN PAGE/immunoblot was performed separately for each subunit. This may explain the apparent size variation of the complexes on different blots. For example in Fig 1A, the two bands labelled as 920 and 770 complexes appeared to run much higher than what I assumed to be the 720 KDa reference band (not labeled) in the TAC60-myc blot, as compared to these bands in the TAC40 and TAC42-HA blots.*

The same markers were used for all lanes in Fig. 1B. The molecular weights corresponding to the various marker band is only indicated on the left most panel.

The lanes are from different 4-13% gradient BN-PAGE gels. The slightly different migration patterns on the different lanes can be explained by small variations when pouring gradient gels. Moreover, due to “smiling” effects, there can also small variations between different lanes on the very same BN-PAGE gel. However, the molecular weights of the indicated complexes was individually calculated for each lane using the linear models depicted in Fig. S5, which show a strong correlation of the “relative migration distance of marker proteins” with “the square root of the molecular weight in kDa”. Using this information the molecular weight of the subcomplexes, including the 3xHA or 3xmyc tags were applicable, have been estimated to be 770 kDa and 920 kDa within a variation of < +/-15 kDa.

*Considering the variation in BN -PAGE results, and the importance of accurate subcomplex identification in this study, I would suggest expressing TAC42-HA and TAC60-myc tags in the same cell line, and probe for all 3 subunits (with different antibodies) on samples fractionated on the same BN-PAGE gel.*

We have observed in the context of other projects (such as the characterization of the ATOM and the TIM complexes), that expression of two tagged subunits of the same complex can be difficult. The reason is that when both the tagged and the untagged subunits are expressed in the same cells, the cells normally integrates the untagged subunits into the complex. – A way around this, would be to use a cell line that exclusively expresses the tagged variants of TAC42 and TAC60. However, considering the limited numbers of resistance markers available for *T. brucei*, to produce such cell line very difficult and time-consuming. We would need to first establish a TAC42/TAC60 double RNAi cell line and then re-express the two tagged variants of the proteins.

*3. The presentation of Fig. 5 is confusing. Based on the gel/blot background, the NYsm sample (dotted lines) seems to be taken from a BN-PAGE experiment different to the other 3 samples. However, all 4 lanes are in the same solid line box and share the same molecular weight standard.* *It seems that the myc tag on TAC40 significantly increased the size of each of the subcomplexes? Please clarify this point. Besides, was the blot probed with anti-TAC40?*

All lanes of Fig. 5 are adjacent to each other and stem from the very same BN-PAGE gel. The broken line after NYsm lane should indicate that it was exposed differently than the three remaining lanes (see Fig. 1 below, which shows the same gel with equal exposures of all lanes). The unequal exposure parameters are now mentioned in the legend of Fig. 5 in the revised manuscript (lines 396-398).

Yes, the 3xmyc-tag in this TAC40-myc cell line does significantly impact the migration distance. Please note that this cell line exclusively expresses the tagged TAC40. - Yes, the blot was probed with anti TAC40 antiserum which recognizes both tagged and untagged TAC40. This is indicated at the bottom right of the gel. Using the antiserum against the untagged TAC40 shows that no wild-type TAC40 is present in the 2 hours induced TAC40 double knock out cell line, as would be expected.

Figure 1 (for reviewers only)

The figure shows an image of the immunoblot shown in Fig 5 acquired with identical sensitivity and exposure for all lanes.

*Based on Fig. S3, ani-TAC40 has a nonspecific band in denatured samples. Nonspecific label seems absent under native conditions, at least for the BSF samples shown in Fig. 5. But how about PCF cells? Could any of the TAC40-containing subcomplexes observed in Figs 1-4 be caused by non-specific labelling? Think this is an important control to include.*

This requested control has now been included in the revised manuscript as new Fig. S2.

**Reviewer #2:**

*Reliance on a Single Methodology: The majority of the conclusions rely on BNPAGE analyses. Incorporating additional techniques, such as mass spectrometry of affinity-purified complexes or reconstitution experiments in a heterologous system, would significantly strengthen these findings.*

Previous publications from our lab included individual proteomic analyses of TAC40, TAC42, TAC60, TAC65 and pATOM36 SILAC pulldown experiments [36, 48]. Prompted by the reviewer comments we have now integrated all five data set and quantitatively analyzed them together. This allowed the construction of an edge-weighted protein-protein interaction network visualizing the digitonin-soluble interactomes of TAC40, TAC42, TAC60, TAC65 and pATOM36 (new Fig. 1A). The results of these analyses confirm i) the proposed two subcomplex classes, and ii) provides strong evidence that the two subcomplex classes consist only of TAC40/TAC42/TAC60 and TAC65/pATOM36, respectively, and do not contain any other proteins in stoichiometric amounts.

A new paragraph describing these findings has been added (lines 152-163):

“To analyze the pull-down experiments in more detail, we pooled all five data sets to generate an edge-weighted protein-protein interaction network visualizing the digitonin-soluble interactomes of TAC40, TAC42, TAC60, TAC65 and pATOM36 (Fig. 1A). This network confirmed the two previously suggested soluble OM TAC module subcomplex classes mentioned above (Fig. 1A, blue and red). Notably, there are no connections between the two subcomplex classes, except for a non-reciprocal very low enrichment of TAC60 in the pATOM36 pulldown. The only notable additional proteins recovered in more than one pulldown experiment are POMP19 and J31, subunits of a quality control pathway connected to α-helically anchored OM proteins, and an uncharacterized metallopeptidase (Tb927.9.13490), a known contaminant detected in previous unrelated pulldown experiments.”

The “Figure legends” and the “Material and Method” section have been changed accordingly

Reconstitution experiments in heterologous systems would indeed be a nice complementary approach. However, we believe it is beyond the scope of the present study. It should be considered that the two subcomplex classes we studied consist of integral membrane proteins which in our experience would make this approach very challenging.

*Functional Clarity: The precise role of the identified TAC subcomplexes remains unclear. Clarifying their functional relevance would greatly enhance the significance and impact of this study.*

The present study is, to our knowledge, the first one providing an experimentally supported model of the assembly process of the OM TAC module of the TAC. However, having more information regarding the precise functional significance of the various subcomplexes would of course be most welcome.

In order to provide a deeper analysis of the assembly of the TAC40-, TAC42- and TAC60-containing subcomplexes, we expanded the experiments shown in Fig. 4A of the original manuscript. Instead, of only providing a qualitative analysis of the formation of the TAC40-containing subcomplexes we now performed time course experiments which provide additional insights into their assembly kinetics. The experiments were preformed in triplicate and were statistically analyzed (new Fig. 4A). The results support the assembly model of the TAC40-, TAC42- and TAC60-containing subcomplexes that is proposed in Fig. 8B.

A new paragraph describing these findings has been added (lines 316-334):

“Instead, we saw a time-dependent accumulation of the two TAC40-, TAC42-, and TAC60-containing subcomplexes (~770 and ~920 kDa). Intriguingly, it appeared that the ~770 kDa subcomplex accumulated faster than the ~920 kDa subcomplex and that its levels declined at later time points of induction. This was not just the case for the mean values of the triplicate experiments (Fig. 4A, left graph) but the same was also seen in each of the three individual experiments included in the analysis. However, the levels of the smallest subcomplex of ~535 kDa consisting of a TAC40 oligomer remained constant (Fig. 4A, left panel). The same time course experiment was also performed in cell lines simultaneously depleted for both the cytosolic and inner TAC modules and similar kinetics were observed (Fig. 4A, right panel). Although in this case it was less clear whether the ~770 kDa subcomplex accumulated faster than the ~920 kDa subcomplex. These results were consistent with the notion that assembly of the TAC40-, TAC42- and TAC60-containing OM TAC module subcomplexes started with ~535 kDa oligomer that after addition of TAC42 and TAC60 was first converted into the ~770 kDa and later into the ~920 kDa subcomplex.

However, there are some caveats to be considered in the interpretations of the experiments shown in Fig. 4A. Comparisons of the data points from triplicate experiments before and after RNAi induction using ANOVA followed by *post hoc* Student’s t-tests and the Holm-Bonferroni method resulted in adjusted *P* values <0.1 but did not always reach the statistical significance of <0.05. Nevertheless, because the same quantitative trends were observed in both sets of experiments (n=3 each) done in two different cell lines (Fig. 4, left and right panels) this strongly suggests that the data at least globally represent the in vivo assembly kinetics of the corresponding OM TAC module subcomplexes.”

The “Figure legends” and the “Material and Method” section have been changed accordingly.

Moreover, we have also used a novel experimental approach, that is independent of all other presented experiments in the study, to test the proposed assembly model. We overexpressed TAC60 and found that this induces a large change in the steady state levels of all three TAC40-containing subcomplexes. The way the levels change is consistent with our assembly model for this class of subcomplexes.

The new results are shown in new Fig. 4C and described in a new subchapter.

“**TAC60 overexpression changes the levels of the TAC40-containing subcomplexes** (lines 347 –359)

To test the stepwise assembly model outlined in the previous paragraph we used a cell line allowing ectopic tetracycline-dependent expression of TAC60-myc in a wild-type background. The results in Fig. 4C shows that after addition of tetracycline, and thus overexpression of TAC60-(myc), the abundance of the ~535 kDa TAC40-oligomer was much reduced, whereas the level of the largest ~920 kDa TAC60-containing subcomplex increased. Moreover, the levels of the ~770 the kDa subcomplex stayed the same and an additional high molecular weight subcomplex of ~1000 kDa, that was not seen in the absence of tetracycline, was also detected. The simplest explanation for these results is that the higher level of TAC60-(myc) caused a more efficient conversion of the ~535 kDa TAC40 oligomer either directly, or more likely via the ~770 kDa subcomplex, into the ~920 kDa subcomplex and finally even into the yet uncharacterized ~1000 kDa subcomplex. It also suggests that the abundance of TAC60 is a rate-limiting factor for the formation of the ~920 kDa subcomplex.”

The “Figure legends” have been changed accordingly and a short paragraph has been added to the “Discussion” section (lines 540-542).

**Part III – Minor Issues: Editorial and Data Presentation Modifications**

*Please use this section for editorial suggestions as well as relatively minor modifications of existing data that would enhance clarity.*

***Reviewer #1****:*

*1. As BN PAGE results may vary greatly between experiments and gel conditions, the BN PAGE methods should be described in greater details. Is there a reference for the BN-PAGE protocol used in this study? What was the buffer used to prepare for the 4-13% gel? What was the running buffer? And the running conditions?*

A more precise description of the BN PAGE methodology used in the manuscript has been added to the Material and Methods section of the revised manuscript (lines 670-681).

*2. Lines 242-243. Depletion of either pATOM36 or TAC65 disrupted the 440 complex. This is not unexpected for protein complexes, where depletion of a subunit leads to the dissociation of the entire complex. This however, does not mean that the 440 complex contains only these two subunits (as concluded by the authors).* *The presence of other subunits in this complex cannot be excluded based on this experiment alone.*

We now provide an edge-weighted protein-protein interaction network visualizing the digitonin-soluble interactomes of TAC40, TAC42, TAC60, TAC65 and pATOM36 (new Fig. 1A). This interaction network is based on previously performed proteomic analyses of SILAC pulldown experiments of all five OM TAC module subunits [36, 48]. The results of these experiments strongly suggest that no other proteins are stoichiometrically associated with the corresponding subcomplexes.

For a detailed discussion of the results and the modification that were made to the manuscript, see response to the first comment of reviewer 2 (Part II, major issues).

*3. Line 95. “a few thousand copies have [been] identified so far…”*

Corrected (Line 91)

*4. The 535KDa subcomplex was found to be TAC40 oligomer (lines 237-238). Could the authors explain a bit more about this? The 535 subcomplex could be labelled by anti-TAC40, but not by anti-myc or anti-HA (for TAC60 and TAC42 respectively), but other protein subunits could still be present in this subcomplex?*

See response to point 2 above

***Reviewer #2:***

*Figure 1B: It is unclear whether the molecular mass markers differ between the two blots presented.*

(now Figure 1C) Yes - the molecular mass markers differ between the two blots. This is why the molecular weight corresponding to each marker band is individually indicated.

*In the absence of mass spectrometry for affinity- or immuno-purified complexes separated by size, the conclusion that the 535 kDa complex consists solely of TAC40, and the 770 kDa and 920 kDa complexes contain varying amounts of TAC42 and TAC60, is speculative. This conclusion needs further support beyond BNPAGE results.*

See response to point 2 above.

*Additionally, the authors should emphasize that co-immunoprecipitation (co-IP) indicates protein interaction but does not confirm direct protein-protein interactions.*

We added the following sentence at the beginning of the “result section” (lines 161-163):

“We would like to emphasize that pull down experiments allow to define the composition of protein complexes but do not provide information which subunits directly interact with each other.”

*Furthermore, RNAi efficiency should be explicitly assessed and presented.*

The RNAi cell lines in question have previously been characterized and published (see Table 1). We used liquid N_2_-preserved stabilates of previously established cell lines, growth defects and kDNA loss were confirmed in every case.

*Figure 2: Clarify the meaning of the '+' and '–' symbols in the figure; presumably, these refer to RNAi induction status.*

The ‘+’ and ‘-‘ stand for the presence (‘+’) and absence (‘-‘) of the indicated protein(s). The reason for this notation is that depending on the cell line (RNAi cell line vs. conditional knock down cell line) the presence and absence of tetracycline correlates directly or inversely with the presence and absence of indicated TAC subunits. The chosen notation is consistent throughout the manuscript.

*Additionally, specify the duration of RNAi induction and quantify the efficiency of targeted protein depletion.*

This information has been added at the top of the chapter “Cell fractionation” in the “Material and Method” section (Lines 649-652). See also response to similar request above.

*The appearance of a TAC42-containing smear in the TAC40 and TAC60 RNAi conditions appears to be overstated and needs cautious interpretation.*

The phrase in question has been toned down (Line 222)

*Figure 7A: Mass spectrometry analysis of the pull-down complexes would substantially reinforce the proposed model of complex architecture and provide direct evidence supporting the subcomplex formation*

See response to point 2 above.

***Reviewer #3:***

*I have only a few comments to strengthen the paper:

  Following the different TAC 'a', 'b', 'c', etc… assembly descriptions can be challenging. Readers would benefit from Fig S1 (overall TAC strucrure) being presented up front, as a main figure.*

We decided against this because PLoS Path is a online journal. Thus supplementary Figures can be as easily accessed as main Figures.

*The use of alpha fold to predict potential structural arrangements of TAC40 within the TAC40 oligomer is presented without a clear rationale for why it might be important or informative to have these models. Is there something known regarding AA subunits that interact with other subcomplexes, thus making knowledge of TAC40 oligomer structure important for understanding subcomplex interfaces? Are there specific predictions based on the candidate structure model that can be tested? It seems this data is extraneous to the main thrust of the paper.*

In our study we define the composition of the various TAC subcomplexes. The AlphaFold predictions, especially the one of the TAC40 oligomer, indicate that these compositions are consistent with structural predictions. Furthermore, we use AlphaFold predictions specifically to estimate the number of TAC40 subunits in the TAC40 oligomer.

*Cytoplasm vs Cytosol:  throughout the MS the authors use the term "cytosol", which is a biochemical description for soluble material within the cytoplasm, when i think they mean "cytoplasm", the compartment  comprising the cell interior. Please consider replacing the term accordingly.*

We believe the term “Cytosol” is correctly used, because it excludes the mitochondrion as intended.

https://www.labxchange.org/library/items/lb:LabXchange:93419a4c:html:1
